# Reply to: Comments on “Association between subjective physical function and occurrence of new fractures in older adults: A retrospective cohort study”

**DOI:** 10.1111/ggi.14919

**Published:** 2024-06-07

**Authors:** So Sato, Yusuke Sasabuchi, Shotaro Aso, Akira Okada, Hideo Yasunaga

**Affiliations:** ^1^ Department of Clinical Epidemiology and Health Economics, Graduate School of Medicine The University of Tokyo Bunkyo‐ku Japan; ^2^ Department of Real‐world Evidence, Graduate School of Medicine The University of Tokyo Bunkyo‐ku Japan; ^3^ Department of Prevention of Diabetes and Lifestyle‐Related Diseases, Graduate School of Medicine The University of Tokyo Bunkyo‐ku Japan

Dear Editor,

We appreciate the comments by Togashi *et al*.^1^ on our article.^2^ In response, we provide further information and perspectives on these points.

First, our study's primary focus was to evaluate the predictive ability of the three items related to subjective physical function, because it remains unclear whether these three items are associated with the occurrence of new fractures. Future studies could incorporate analyses of all 15 items to compare their predictive abilities.

Second, as mentioned in the limitations of our study, past and new fractures were not clearly distinguished.^2^ Thus, we conducted a Cox regression analysis similar to the main analyses wherein participants with past fractures or osteoporosis were excluded. The results showed new fractures were associated with subjective gait speed decline (hazard ratio 1.55, 95% confidence interval [CI] 1.11–2.17), recent fall history (hazard ratio 1.39, 95% CI 0.97–2.00) and the absence of exercise habits (HR 1.28, 95% CI 0.93–1.76). These results were consistent with those of the main analyses, although the CIs were wider because of the smaller sample size.^2^

Third, the database lacks traceability of individuals before and after their subscription to the Late Older Medical Service System. However, this lack of traceability was not significant in our study. We primarily focused on older adults aged 76 years using a 1‐year look‐back period before the index date, as enrollment in the Late Older Medical Service System typically starts at the age of 75 years.

## Funding information

This work was supported by a grant from the Ministry of Health, Labor and Welfare, Japan (23AA2003).

## Disclosure statement

The authors declare no conflict of interest.

## Ethics statement

This study was approved by the Institutional Review Board of the Graduate School of Medicine at the University of Tokyo (approval number: 2021010NI [23 April 2021]). The requirement for written consent was waived owing to data anonymity.

## Data Availability

The data that support the findings will be available in DeSC Healthcare, Inc at https://desc-hc.co.jp/contact following an embargo from the date of publication to allow for commercialization of research findings.
